# Enhancing Resilience in Community-Dwelling Older Adults: A Rapid Review of the Evidence and Implications for Public Health Practitioners

**DOI:** 10.3389/fpubh.2019.00014

**Published:** 2019-02-07

**Authors:** Wendy Madsen, Meghan Ambrens, Melanie Ohl

**Affiliations:** School of Health, Medical and Applied Sciences, Central Queensland University, Rockhampton, QLD, Australia

**Keywords:** resilience, community, adversity, aging, volunteering, resources

## Abstract

**Background:** Resilience is a valuable platform for strengthening individuals and communities in the face of disaster. This review sought to evaluate the current literature related to individual and community resilience in community-dwelling older adults to understand the status of resilience in this population, identify gaps, and make recommendations about effective interventions that promote improved individual and community level capacity. Recognizing the concept of resilience is contested, the review conceptualized resilience as a collective concept that is multi-level and interdependent across those levels, cumulative, and contingent on context.

**Methods:** The rapid review located 29 relevant peer review journal articles and industry reports related to research or evaluations of interventions aimed at increasing resilience at either a personal or community level. The results of these papers were thematically analyzed.

**Results:** This review found personal resilience relates to those personal capacities and resources one has and uses to deal with individual stresses and change. We identified several recurring themes within those studies focused on personal resilience, including: (1) positive reframing and agency; (2) personal meaning and purpose; (3) acceptance and belonging. At a community level, we identified the following themes influence collective capacity and resources: (1) empowerment and shared decision making; (2) collective agency; and (3) collective leadership and engagement.

**Conclusions:** The review highlighted the need to reframe how communities view older adults and shift the narrative away from focusing on age-related deficits toward acknowledging the economic and social contribution older adults make to the community through activities such as volunteering and the sharing of knowledge of history, culture and skills. Demonstrating the interdependence across levels, these activities illustrate personal-level capacities promoting collective action and participation as important for increasing community resilience. The review argues resilience is developed in everyday circumstances, therefore active involvement within communities needs to be encouraged within community-dwelling older adults. Developing active involvement will not only contribute to both personal and community level resilience but will enable communities to prosper and flourish through adversity.

## Introduction

### Rationale

Australia has a long history of being impacted by natural disasters which can have a devastating impact on individuals and communities. Resilience is emerging as a valuable platform for strengthening individuals and communities in the face of disaster and change (1). Resilience is conceptualized here as the ability to withstand, adapt and transform capacities and resources in the context of uncertainty, change, unpredictability and surprise (1, 2). This conceptualization of resilience moves beyond the individual notion of bouncing-back, to a collective concept that is determined by interdependent factors such as community resources, social and economic capital, and governance (1). In addition to facilitating resilience within the wider community, resilience in older adults can be an important determinant of healthy aging (1).

Resilience is a term that has been used by a variety of disciplines over a number of decades. As such, resilience has come to have different meanings, and is a highly contested concept within the literature. Thus, there are numerous literature reviews defining and conceptualizing resilience, for example, Castleden et al. (3), Norris et al. (4), and Reid and Courtenay Botterill (5). Skerratt (6) outlined a typology of resilience from the 1940s to 2010, indicating three main areas of focus: physical systems (including maths, ecological systems, and psychological research); social-ecological systems; and human agency systems. The first two areas of focus conceptualized resilience as a way of bouncing-back from external shock or disaster, while the third focus took a different approach to conceptualize resilience in terms of proactive agency in a context of constant change, mechanisms, resources and vulnerabilities. While there has been some convergence in resilience thinking over the past decade (7), a number of authors suggest reaching consensus is not necessary, and that it is resilience's fuzziness and versatility that has the potential to establish it as a “shared, multi-sectoral framework for building flourishing communities” [(8), p. 363]. This means it is important that each study of resilience clarify the particular conceptualization of resilience used.

The points of convergence regarding resilience include an understanding that resilience is:

- Multi-level (includes individual, community, and regional levels) and interdependent across these levels;
- Cumulative (built over time through repeated mechanisms and pathways); and
- Contingent on context (social, cultural and material resources and ways of doing, being, and knowing) (6, 9, 10).

Some salient points that continue to be contested include whether resilience is a trait, process, outcome or the source of an outcome (6, 9, 11). This lack of agreement has particular political implications when resilience is applied as a policy to encourage local communities to address their own challenges. Steiner and Markantoni (10) urge researchers and practitioners to take a critical perspective of government policies that aim to “empower” local communities under the banner of building resilience while at the same time withdrawing resources and services.

In outlining the conceptualization of resilience for public health, Seaman et al. (1) highlight the need for resilience thinking across culture, economics, governance, and infrastructure at both individual and community levels. For the purposes of this paper, Magis' (2) definition of community resilience has been drawn on as it incorporates: (1) both individual and community levels; (2) process, resource, and outcome aspects; and (3) acknowledges the importance of context. It is a proactive agency perspective rather than a neutral conceptualization that places value on resilience as a common “good.” This coincides with health promotion objectives of wellbeing and flourishing (1).

*Community resilience is the existence, development, and engagement of community resources by community members to thrive in an environment characterised by change, uncertainty, unpredictability, and surprise. Members of resilient communities intentionally develop personal and collective capacity that they engage to respond to and influence change, to sustain and renew the community, and to develop new trajectories for the communities' future* [(2), p. 402].

Magis' (2) definition falls within Skerratt's (6) typology at the human agency systems end of the spectrum. It has also remained relevant; as concepts of resilience have developed in recent years Magis' (2) definition has been the basis of several resilience studies, including those identified for this rapid review (12–14). This definition is also broad enough to cater to a variety of circumstances including rapid and slow-moving natural disasters such as tropical cyclones and drought, as well as change and uncertainty that is influenced by human systems such as economic crises and food or water insecurity.

**Figure 1:**
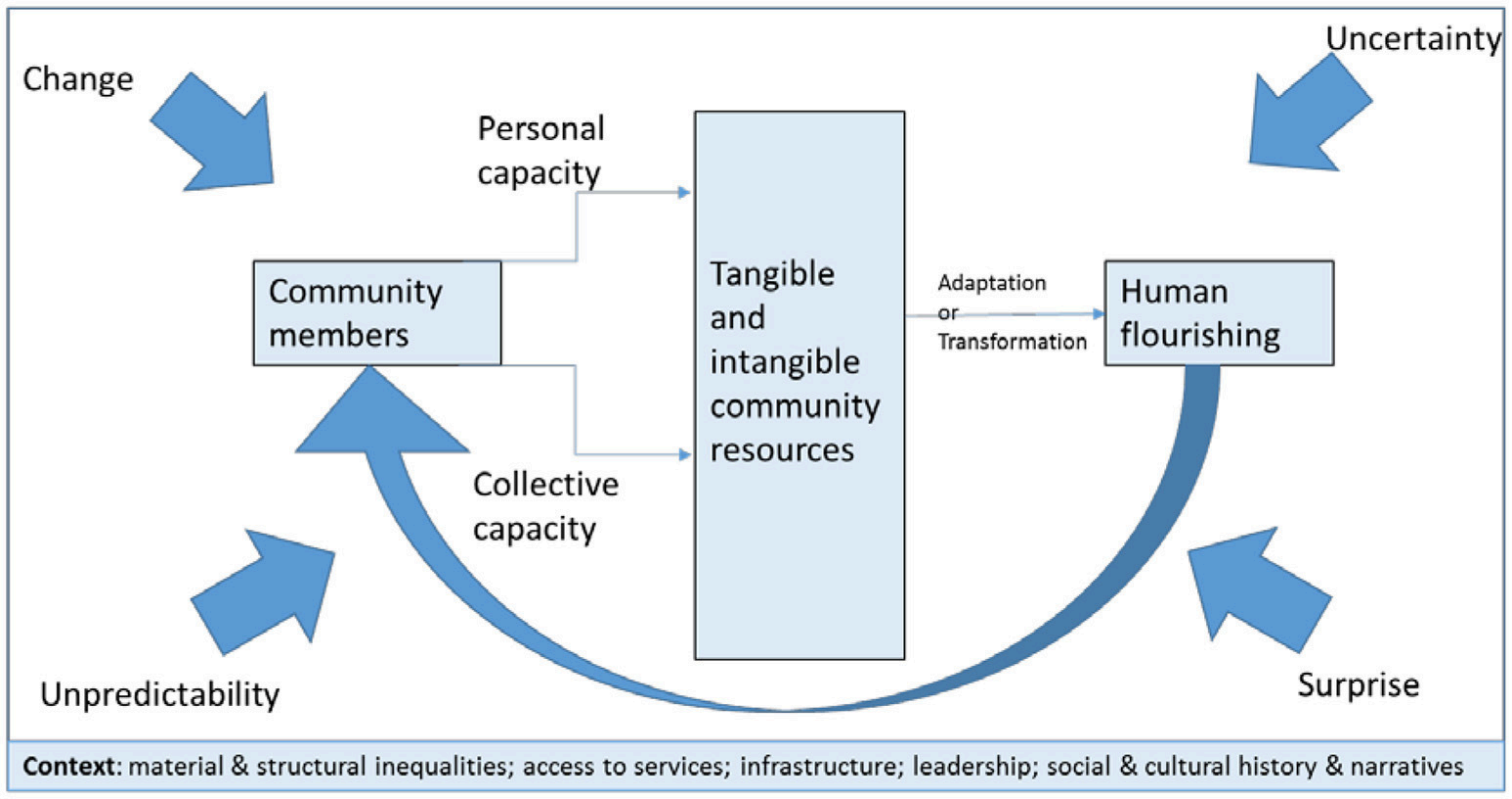
Conceptual framework of resilience.

Magis' (2) definition of resilience, in conjunction with the work undertaken on resilience for public health by Seaman et al. (1), provides the conceptual framework for this rapid review (illustrated in Figure 1). This conceptual framework facilitated the focus on aspects of resilience amenable to community interventions that can be undertaken either as part of a natural disaster recovery program or general community development activities. Specifically, interventions aimed at developing personal capacity, collective capacity, or tangible and/or intangible community resources that allow communities or groups within communities to adapt successfully to change or to transform the context itself in some way to sustain human wellbeing are supported within the framework. In addition, understanding the material and social inequalities that may characterize any particular community or context must be taken into account when considering the people living in that context: their educational and economic opportunities; their cultural connections and practices; their social and historical sense of belonging; and their social connections within and beyond the community. All of these factors will directly or indirectly impact on personal and community resilience.

While considerable attention has been paid to personal resilience, particularly within children and youth, there is an emerging interest in resilience and older adults (1, 15). Janssen et al. (16) point out that this focus on personal and community resilience within older adults is a counter-narrative to the deficit view of aging; that is, resilience shifts the focus toward the positive effects on health and life satisfaction. However, Wild et al. (15) warn this perspective should never be used as a means of transferring responsibility or blaming victims for deficits or declining abilities that may occur with aging. Janssen et al. (16) understanding of resilience in older adults is consistent with the conceptual framework used here, as a multi-leveled interdependence between personal resources (such as psychological traits) and processes, collective resources and processes, and the context within which these interactions play out. This multi-level understanding is important in guarding against transferring responsibility or victim blaming (15).

Wild et al. (15) note a number of researchers believe resilience is cumulative and that this has important implications for older adults. If there is a cumulative aspect to resilience—a process honed by a lifetime of adaptation to hardship—then how we approach resilience and older adults may provide us with an opportunity to review our understanding of the vulnerabilities of older adults during times of adversity such as natural disasters. This presents two implications. First, older adults often have a deep understanding and sense of connection to place. They have stories of how the community has responded to adversity in the past and how the community has rallied. This can be an important source of encouragement and hope. Second, rather than seeing older adults as vulnerable human-beings in need of assistance, the resilience literature emphasizes “the *experience of* rather than the *avoidance of* vulnerability” [(15), p. 142]; that adversity can provide an opportunity to learn and thrive, and that older members of our communities can contribute to this. This is not to suggest that older adults should not be supported by agencies, services, and other community members as needed during periods of crises. Rather, it suggests that if we look at resilience from a broader perspective, older adults have much to offer each other, themselves and the community in encouraging resilience. For example, Skinner et al. (17) outline how the volunteering of older adults in rural communities in Canada has on occasion led to the retention of local services and contributed to economic initiatives. Thus, older adults contribute to their own resilience and that of their communities through their stories, interactions and relationships, and provide leadership and economic benefits through activities such as volunteering. While they may make disproportionate use of various services within communities, older adults are simultaneously ensuring these services are available to all within the community. This latter aspect is particularly important in rural and regional areas that risk having services withdrawn.

### Research Question

This rapid review focuses on community-dwelling older adults, who have traditionally been perceived as vulnerable in times of disaster and change. Therefore, this rapid review seeks to understand the current status of the evidence related to individual and community resilience within community-dwelling older adults by reviewing studies that effectively enhance individual and community resilience in community-dwelling older adults.

### Objectives

The objective of this review was to examine the current body of knowledge to identify and evaluate the effectiveness of resilience-enhancing approaches, and use these findings to inform policy and program development. The review identifies commonalities across resilience-enhancing approaches that have evidence of effectiveness. Such evidence should enable service organizations, agencies, and governments to enhance the effectiveness of their programs and services.

## Methods

### Study Design

This rapid review undertook a review of the peer-reviewed and gray literature to identify evaluation and research studies that capture resilience within regional, rural or remote areas of Australia and internationally, particularly interventions designed to enhance personal and community resilience within community-dwelling adults who are semi-retired or retired and living in good health with no major cognitive, psychological, physical, or clinical conditions.

### Review Protocol

Before commencing this rapid review, a research protocol was developed to ensure our searching parameters were relevant, accurate and methodical. The research question, aims and objectives, study design, definitions and boundaries, inclusion/exclusion criteria and search strategies were clearly outlined within the protocol. This guided the research process and was an effective collaboration tool for reviewers to discuss findings and ensure consistency throughout the process. As new findings were presented, the scope of the project did evolve which was continuously reflected within the protocol. Thus, the protocol identified the inclusion criteria that guided the search strategy and data extraction processes. These inclusion criteria were:

- Original research;
- Primary research or evaluation study;
- Published after 2013;
- Written in the English language;
- Peer-reviewed journal article OR gray literature available from .gov or .org domains;
- Identify interventions which aim to enhance resilience;
- Report outcomes of personal resilience among community-dwelling adults aged ≥50 years of age with moderately good health (see aims/objectives) OR report outcomes of collective resilience across community groups including older adults.

### Search Strategy

To ensure all peer-reviewed research on resilience was located, a comprehensive search of the following databases was undertaken: CINAHL, PubMed, Medline, PsychInfo, PsychArticles, The Cochrane Library, Science Direct, Sociology Source Ultimate, JBI, Scopus, Prospero, Public Health, Academic Search Ultimate, Health Business Elite, Health Management. In addition, to satisfy an exhaustive search, the gray literature websites with .gov and .org domains were searched.

Initially, preliminary searches were conducted to test search term accuracy. Boolean logic was applied where possible to enable breadth across searches. Once key terms were finalized, two independent searches were performed to explore both personal and community concepts of resilience within the target group. The search term strategies are identified in Table 1.

**Table 1:** Search terms.

**Personal resilience search terms**	**Community resilience search terms**
1. Resilien^*^ 2. AND personal capacit^*^ OR hope OR agency OR motivation OR optimism OR self-efficacy OR positive emotion 3. AND intervention OR program OR strateg^*^ OR project 4. AND communit^*^ dwelling OR independen^*^ 5. AND senior OR aged OR older adult OR older OR elderly OR 50 years 6. AND regional OR rural OR remote	1. Community resilien^*^ 2. AND collective capacit^*^ OR social empowerment OR social capital OR sense of community OR collective efficacy OR network 3. AND intervention OR program OR strateg^*^ OR project 4. AND communit^*^ dwelling OR independen^*^ 5. AND senior OR aged OR older adult OR older OR elderly OR 50 years 6. AND regional OR rural OR remote

### Data Sources, Studies Sections, Data Extraction

**Figure 2:**
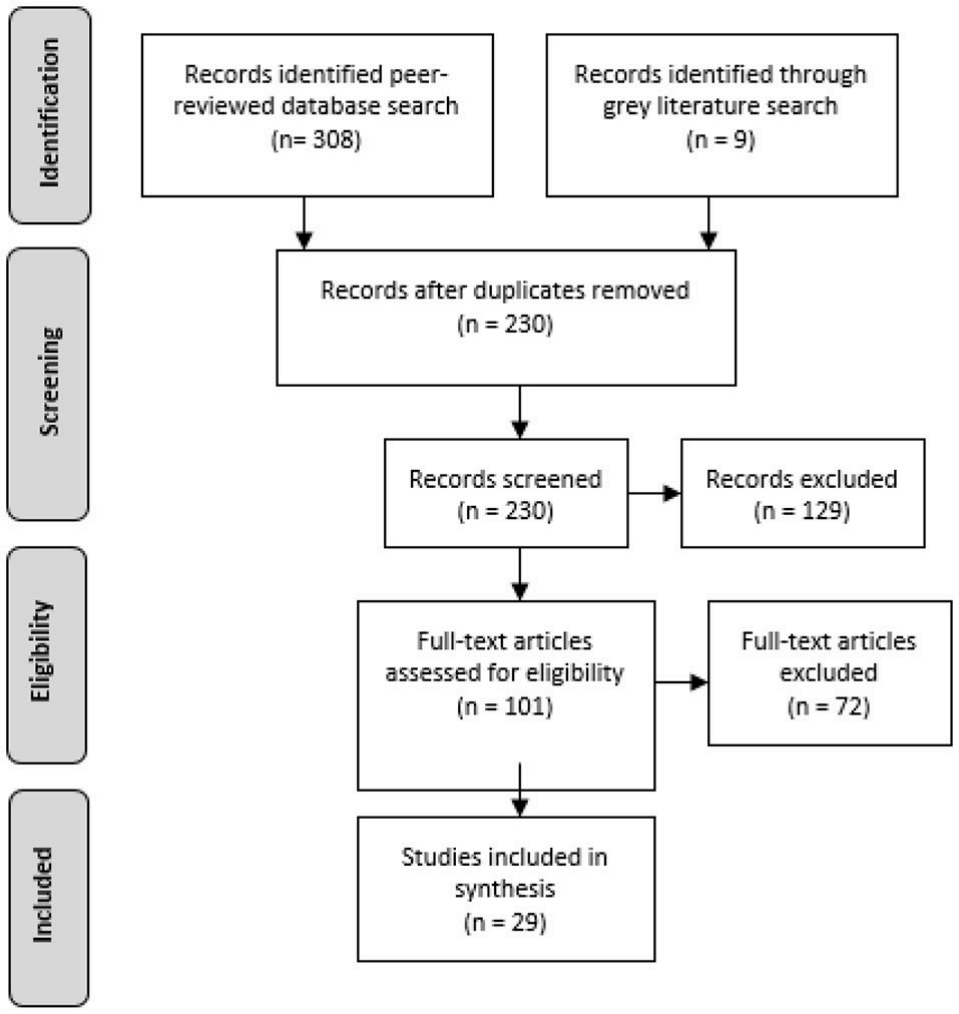
Literature screening matrix.

In total, 308 peer-reviewed articles were retrieved from the database searches and 9 studies retrieved from gray literature searches in accordance with study title relevance (see Figure 2). Once duplicates were removed, the abstracts of 230 studies were screened and 129 articles were excluded based on not meeting the inclusion criteria (for example, focused on children or a cohort with significant mental health issues). A screening tool template was then developed so that the full text of 101 articles could be assessed for eligibility. Based on the inclusion criteria set out in the screening tool, 29 resilience-focused studies were identified for inclusion.

### Data Analysis

Together, three reviewers used data extraction tables to explore the key concepts of each study, including their sample size, target groups, country of focus, study quality, and main outcomes/findings (See Tables 2, 3). The main outcomes and findings were thematically analyzed.

**Table 2:** Summary of reviewed papers—personal resilience.

**Paper and Country/Target group**	**Years**	**Aim**	**Population**	**Data collection and analysis**	**Results**
Nitschke et al. (18) *Australia*	2013	Explore resilience, behaviors, health risk factors and health outcomes during heat waves independently living residents	*N* = 499 (aged >65 years; *n* = 341 from metro, *n* = 158 rural)	Random telephone survey	75% of older adults undertook adaptive behavior during heatwaves, although were not informed or did not see themselves at risk. Notes importance of social connectedness in reducing risk of mortality during a heat wave.
Severinsen et al. (19) *New Zealand*	2016	Examine the narratives older people use to describe their housing preferences in later life	*N* = 143 (aged 65–93 years)	In-depth interviews Narrative analysis	Acknowledged older adults have a preference to remain in own homes as they age, although housing and/or location may become unsuitable for their changing needs Attachments to home and local community and feeling “in-place” improves well-being.
Annear et al. (20) *New Zealand*	2014	Develop community-generated recommendations to inform urban environmental remediation following earthquakes suitable for independently living older adults	*N* = 38 (aged ≥65 years; *n* = 30 volunteers; *n* = 8 knowledgeable advisers)	Focus group discussions to critique mixed-methods and multiphase results	Developed 6 recommendations and associated actions: 1. Remediation of earthquake damaged areas 2. Transport and mobility needs 3. Age-friendly rebuilding 4. Safer communities 5. Resilient support agencies 6. Social and cultural venues.
Baldacchino et al. (21) *Australia and Malta*	2014	Detect any significant differences in spiritual coping strategies of older people related to their personal characteristics	*N* = 137 (mean age = 72.8 years; *n* = 103 men, *n* = 34 women; *Australia*, private homes *n* = 30; *Malta*, private homes, *n* = 43; Malta, residential, *n* = 64)	Descriptive sequential explanatory study Phase I: self-administered questionnaire and the Spiritual Coping Strategies (SCS) scale	Maintaining relationships with friends, family, room-mates, God and nature lessened anxiety and depression, and helped older adults adapt to their current situation. Spiritual coping is higher in those living in private homes in both Malta and Australia, although culture plays an important role in spiritual coping.
Baldacchino et al. (22) *Australia and Malta*	2014	Explain the impact of the use of spiritual coping strategies on institutionalized older persons	*N* = 137 (mean age = 72.8 years; *n* = 103 men, *n* = 34 women; *Australia*, private homes *n* = 30 *Malta*, private homes, *n* = 43; Malta, residential, *n* = 64)	Descriptive sequential explanatory study Phase 2: audiotaped face-to-face interviews (*n* = 42) Phase 3: three focus groups (*n* = 23)	Three main themes: 1. self-empowerment through connectedness with God, self, others and nature; 2. belongingness to the residence/institution; 3. the finding of meaning and purpose in life or the perceived after-life.
Banbury et al. (23) *Australia*	2017	Examined the relationship between changes in social support networks for older people living in a regional area following weekly videoconference groups delivered to the home	*N* = 52 (Mean age = 73 years; *n* = 25 female; *n* = 27 male)	Semi-structured interviews and focus groups	As people age in their homes, there is a greater risk of social isolation, which can be ameliorated by support networks with health professionals, family and friends. Videoconference education groups delivered into the home can provide social support and enhance self-management for older people with chronic conditions.
Bei et al. (24) *Australia*	2013	Examine the impact of floods on the mental and physical health of older adults and explore the risk and protective factors	*N* = 274 (Age >60 years; 147 were lost to follow up/dropped out)	Longitudinal prospective design Pre- and post-surveys (a flood event)	Floods did have an adverse impact, especially amongst individuals with greater flood exposure and inadequate social support. Includes higher PTSD and anxiety symptoms, but not higher rates of depression. Acceptance, positive re-framing and humor found to be protective factors.
Bellamy et al. (25) *New Zealand*	2014	Explore older adult's views, experiences and sources of bereavement support following the death of a spouse, family member or other significant individual	*N* = 28 (aged ≥75 years; 65+ for Māori participants)	Telephone interviews Grounded Theory	Four main themes were identified: 1. Equanimity and resilience; 2. Older peoples' views and experiences of formal bereavement support services; 3. The pivotal role of family and friends in the provision of bereavement support; 4. The value of harnessing support from existing community and religious organizations in ensuring adequate support for older bereaved adults.
Gibb et al. (26) *Australia*	2018	Investigate how people managed to stay resilient as they aged in remote places	*N* = 14 (Age 61–80 years)	Individual interviews Follow-up focus groups	Highlights the importance of place as a set of conditions for sustaining resilience, psychological integration and belonging. Volunteering supplemented gaps in aged support services. Material sustainability and personal care support also noted as important.
Inder et al. (27) *Australia*	2015	Explore relationship between demographic, socioeconomic and mental health factors and personal hopefulness, including the influence of locality and remoteness	2,774 participants (53% female, mean age 69.1 years, 36% living outside metropolitan areas)	Data from two community-based longitudinal cohorts from New South Wales—one urban and one rural	Five factors were independently associated with lower personal hopefulness: being older, having lower perceived prosperity, less frequent socialization, experiencing high psychological distress or psychological impairment. Hopefulness was not associated with geographical location.
Moylan et al. (28) *Australia*	2015	Explore individual and community contribution of Community Men's Sheds (CMS) in terms of health, well-being and spirituality	*N* = 21 men (Varying ages)	Participant observation over a 6-month period and semi-structured in-depth interviews	Benefits provided by CMS: 1. Increased self-esteem and empowerment; 2. Respite from families; 3. A sense of belonging in the community; 4. Opportunity to exchange ideas relating to personal, family, communal, and public health issues; 5. Spiritual support.

**Table 3:** Summary of reviewed papers—community resilience.

**Paper and country**	**Years**	**Aim**	**Population**	**Data collection and analysis**	**Results**
Wood et al. (29) *Australia*	2015	Document and reclaim stories of survival and resilience to enable people to speak of future hopes and dreams	Kalumburu (indigenous) community	Narrative Inquiry using an online outsider witness practice	Strong Women's Group used re-authoring, remembering, outsider witness process and definitional ceremonies to re-frame problems within the community and plan for collective action around: youth crime and detention, lack of child education, alcohol abuse, chronic disease (diabetes and heart disease), neglect, violence and families fighting, suicide of young people.
Cinderby et al. (30) *United Kingdom*	2016	Examine experience to build community resilience, where facilitators supported residents to take ownership of their own agendas	*N* = 249	Participatory Action Research	Highlights importance of improving quality of life and capacity of neighborhoods build resilience and how the community manages its environment. This includes: 1. Transforming social relationships; 2. Strengthening institutions; 3. Influencing local power balances.
Congues (31) *Australia*	2014	Examine the impact of drought on a farming community and success of attempts to promote and empower resilience and social connectedness	Whole community (size not specified)	Interpretive document analysis of range of evaluation reports and minutes	*Strong women, strong families* program identified as very successful (event for rural women to develop sense of friendship, ownership, education on mental health, enable empowerment), although criticized by some as led to belief women were responsible for welfare of husbands. Recommendation similar program be developed for men.
de Schweinitz et al. (32) *Alaska*	2017	Explore perceptions of causes and prevention of suicide and the functioning of Village Wellness Teams within rural Alaska Native community	*N* = 54 (*n* = 43 women; *n* = 11 men)	Focus groups	Participants were willing to directly confront the topic of suicide and its prevention, including the community's capacity to respond to emergencies and the creation of safe, alcohol- and drug-free events. Believed suicide partly attributed to loss of culture, language, and subsistence activities, as well as limited local economic opportunities and services. Recognized different needs for men and women.
Madsen and O'Mullen (33) *Australia*	2014	Evaluation of locally devised and delivered rural leadership programme	*N* = 16	Interpretative case study using semi-structured interviews	Two key themes illustrate how leadership programmes can contribute to the development of community resilience were identified: 1. Self-development (awareness of strengths and weaknesses of self and others, gaining of self-confidence, and emotional intelligence, increased understanding of leadership); 2. Building social capital (interaction between community members, ability to draw from other people's experiences, share knowledge, strengthens bond of community).
Madsen and O'Mullen (34) *Australia*	2016	Residents' perception of community resilience and how community resilience can be enhanced	First workshop, *N* = 18 (*n* = 17 female, *n* = 1 male) Second workshop, *N* = 14 (*n* = 13, female, *n* = 1 male)	Participatory research approach using photo-voice and surveys	Five themes identified as important factors in community resilience: 1. Social connectedness and belonging; 2. Optimistic acceptance of circumstances; 3. Learning tolerance and patience; 4. Learning from the past for the future.
McCrea et al. (35) *Australia*	2016	Test predictability of measures and concepts of community wellbeing and community resilience	*N* = 389	Computer Assisted Telephone Interviews. Path analysis to test model of community wellbeing and resilience	Community wellbeing predicted by community spirit and cohesion; services and facilities; community and social interaction; environmental loading; built environment; personal safety. Community resilience.
					predicted by community decision making and trust; trust in industry decision making. Place attachment found to be separate construct and not a part of either community wellbeing or community resilience
Roberts and Townsend (12) *Scotland*	2016	Develop an understanding of cultural and digital capital to evaluate the contribution of creative practitioners to rural community resilience	*N* = 15	Semi-structured interviews based resilience framework themes	Creative practitioners developed adaptive capacities to compensate for slow and unreliable internet. Their contribution to cultural capital depended on connection to local community. Cultural activity seen as having spillover effects: economic benefits; bringing community together; employment/training; diversity.
Sangha et al. (36) *Australia*	2017	Community perceptions of resilience undertaken in two Northern Australian communities	*N* = 188	Survey, focus groups, semi-structured key interviews Desktop studies	Understanding of natural hazards interpreted in context of other hazards, including colonization and government intervention. There is a mismatch between the expectations of government emergency agencies and local communities related to: feelings of safety; recognition of cultural norms, practices and ceremonies; effect of disasters on physical, spiritual and economic conditions in communities; knowledge and accessibility of emergency plans; recognition of local Aboriginal organizations, local social networks and knowledges; ongoing need to strengthen local capacity; need for economic independence.
Smith and Lawrence (13) *Australia*	2014	Investigate food insecurity during and immediately after a major flood event	*N* = 13	Grounded Theory Semi-structured interviews Policy analysis Media and government literature review	Reliance on supermarkets with little food in supply chains, and limited alternative local food supply avenues. During a flood, there is a focus on supplying food to most vulnerable relying on community resources. Despite many examples of positive “collective community capacity” during the flood event (e.g., flexible and innovative use of personal “networking” to move food from one location to another), numerous challenges related to formal decision making and information-sharing processes.
Steiner (14) *Scotland*	2016	Explore whether facilitated community interventions can empower and develop community resilience	*N* = 30	Semi-structured interviews	Six rural communities were facilitated and provided with funding to undertake a community project: 3 completed; 3 did not in the timeframe. Facilitating factors included: funding availability; facilitator; increasing confidence and networking. Challenges included: lack of sufficient information; consistency of facilitator; insufficient communication; need for more flexibility in timelines; danger of dividing communities.
Tudor et al. (37) *New Zealand*	2015	Understand the role of craft and crafting groups in disaster recovery to help build connection to place and provide avenue for growth	*N* = 32 (*n* = 9 interviews *n* = 5 focus groups)	Semi-structured interviews and focus groups	Five themes identified related to role local organically formed groups of crafters played in providing opportunity for adaptation after a natural disaster: 1. Crafting for recovery and healing; 2. Social connection; 3. Learning and meaning making; 4. Giving to others; 5. A vision for the future.
Williams (44) *Australia*	2013	Understand impact of extreme heat events on services and “at-risk” populations, including older community dwelling adults	*N* = 13 health service providers from 11 rural and remote communities	Semi-structured interviews	Increased use of health services but not as refuges from heat (air conditioned). Noted community members moderated own activities to restrict going outside during middle of day. Potential for older people to restrict social access, and to stay in a single air-conditioned room. Need to check elderly if have air conditioning, able to use it or willing to use it, and if have lack of sensitivity to heat. Noted safety issues related to locking doors and windows reducing housing ventilation.
Linnell et al. (39) *Sweden*	2015	Explore two key areas in crisis management: (a) the role of local communities in crisis preparedness and response, and (b) how to involve the citizens in this task	*N* = 33	In-depth interviews	Seven main themes related to enhanced public resilience: 1. Collaboration: Formal and informal practices; 2. Specific competences and general abilities; 3. Collective efforts and individual self-help; 4. Education and empowerment; 5. Traditional communication vs. digital media; 6. Individual motivation and involvement; 7. Generation and age.
Fois and Forino (40) *Italy*	2014	Understand community resilience processes in ecovillage and analyse how disaster served as window of opportunity for sustainability	*N* = 8	In-depth interviews	Critical of paternalistic, top-down approaches to disaster recovery that focus only on immediate housing needs. Longer term solutions created through local empowerment, participation, transparency, long-term visions and sustainability. Disasters can be an opportunity to transform.
Lyon and Parkins (41) *Canada*	2013	Account for deeper social phenomena such as agency, structure, culture and power in social resilience	*N* = 59	Focused ethnographies: key informants interviews; observations; photographs; secondary sources	Margaret Archer's sociocultural theory helps to better understand panarchy of social ecological resilience theory and adaptive cycles from a social and cultural perspective. Provides a richer and more nuanced understanding of community resilience that is not normative.
Stebbing et al. (42) *Australia*	2013	Describe perceived health effects of water insecurity and understand vulnerability, resilience and adaptive capacity in rural towns experiencing drought	*N* = 13	Focus groups and interviews	Adaptive capacity is enhanced when community takes a bottom-up approach to solving its own problems.
Wickes et al. (43) *Australia*	2015	Identify extent to which pre-flood context influences community resilience in post-disaster context	*N* = 4,403 (wave 3) *N* = 4,132 (wave 4)	Longitudinal survey and census data analysis	No major differences in social capital identified across flooded and non-flooded communities. City, state and federal actions may play a role in generating community resilience, as do community competence and actions.
Williams and Shepherd (38) *Haiti*	2016	Explore ventures initiated by local people in aftermath of earthquake: how they acquired and used resources to facilitate community resilience	*N* = 55	In-depth interviews, observations and secondary sources	Two pathways of alleviating suffering were identified: (1) staged resourcefulness that related more to obligation; and (2) deviant resourcefulness that related more to power and social status.

## Results

### Synthesized Findings

The final 29 articles were further categorized into two groups, either: (1) personal resilience; or (2) community resilience. Magis (2) distinguishes between personal and collective capacities and community resources as key components of community resilience. As such, for simplicity, we present the findings along the rupture lines of personal and collective capacities and resources, yet within the studies we reviewed, such distinctions were not always clear, reflecting the multi-level nature of resilience.

#### Personal Resilience

Personal resilience relates to those personal capacities and resources one has and uses to deal with individual stresses and change. We found a number of recurring themes within those studies focused on personal resilience, including: (1) positive reframing and agency; (2) personal meaning and purpose; (3) acceptance and belonging. Each of these will be explored separately.

##### Positive reframing and agency

While Bei et al. (24) suggest unexpected life events can lead to maladaptive coping and are closely associated with poor mental health, they also note positive reframing and humor are ways to improve an individual's personal capacity to deal with change, including supporting transformative change. Traditionally, age and aging have been viewed adversely and considered an undesirable process of unpreventable physical and cognitive decline. However, positive reframing can be a protective factor and contribute to human flourishing as older adults re-story their lives as part of the aging process. This process requires a level of acceptance of oneself that is aided by a healthy dose of humor. It also requires a certain level of agency. Bellamy et al. (25) contend older adults who develop the agency to draw on their age and life experiences are able to cope with expected and unexpected life events. Developing such agency is empowering in itself. Developing spiritual coping strategies may also contribute to empowerment and reframing the aging process (21, 22).

##### Personal meaning and purpose

A number of the studies identify the importance of maintaining relationships with others as the basis of personal meaning and purpose. Cohabitating adults, healthcare providers, family and friends, and others help older adults find meaning and purpose in life. Supporting this, Baldacchino et al. (22) suggest that older adults value a connectedness with others over a relationship with self. Moylan et al. (28) identify tangible community resources such as infrastructure and facilities which provide a place for interaction and help to facilitate inter-personal reflection which is essential for fostering meaning and purpose, whilst enhancing the ability for community members to connect with the moment, to self, to others, and to their environment. This engenders a sense of belonging in those who live in the community, which is acknowledged by Gibb (26) as necessary for sustaining resilience.

##### Acceptance and belonging

This sense of acceptance and belonging is highlighted as important by several authors. Research by Nitschke et al. (18) and Williams (44) indicate that during a heat event, the adaptive capacity of older adults is hampered by a lack of belonging and acceptance, with many older adults reporting feelings of isolation and a loss of confidence in their ability to call friends or neighbors. Such actions are seen to intensify their health risks. Nitschke et al. (18) concludes that social connectedness reduces mortality during a heat wave. Supporting this, Severinsen et al. (19) and Gibb (26) contend older adults value friendships and social connections with the community, neighbors, and family along with the sense of feeling in place. In fact, this sense of belonging and acceptance not only improves the well-being of older adults but enhances their independence and can reduce the health costs associated with aging (19, 23). Banbury et al. (23) argue a sense of belonging and acceptance derived from social support makes older adults feel more engaged with life. Inder et al. (27) suggest social isolation increases feelings of personal hopelessness, psychological distress and lower perceived prosperity among older adults. Annear et al. (20) explore how tangible community resources such as age-friendly policies and funding, in conjunction with appropriate community facilities and support provided through health agencies and social networks, are critical in providing a sense of acceptance and community cohesiveness. This allows older adults to remain in their homes and stay connected to their community as they age and to draw on intangible community resources such as history, personal and collective memories, and a collective sense of belonging and attachment that in-turn fosters adaptation and human flourishing.

#### Community Resilience

The following describes the commonalities identified that influence collective capacity and resources. We identified a number of recurring ideas: empowerment and shared decision making; collective agency; and collective leadership and engagement present in the literature. As with personal resilience, there is some overlap across the themes, but each will be explored separately.

##### Empowerment and shared decision making

Empowerment and shared decision making have been inextricably linked within the community development literature for some time, so it is not surprising these are strongly identified in studies that consider community resilience (45). Fois and Forino (40) suggest shared decision making and collective leadership that focuses on the long-term impacts of decisions for a community and engendered autonomy, enhances community resilience through local empowerment, participation and transparency. Furthermore, McCrea et al. (35) argue community decision making and trust is a key dimension of community resilience. Communities that foster shared decision making and empowerment are more likely to view adversity as an opportunity for transformation. They are able to draw on intangible community resources such as community cohesiveness, collective history, memories and narratives to support local cultural and social practices, and equip individuals within the community to transform and flourish (29, 36). Shared decision making and leadership is a departure from the common discourse seen with top-down approaches that typically isolate members of the community. Fois and Forino (40) claim shared decision making and leadership leads to re-using community resources and re-storying connection to place.

##### Collective agency

Steiner (14) contends agency is the ability to collectively mobilize leading to increased individual and collective capacity and transformation. Research by Roberts and Townsend (12) and Steiner (14) suggest tangible community resources such as infrastructure and community facilities influence the adaptive capacity of those individuals living in the community. Poor physical infrastructure can inhibit individuals and the community by limiting social and cultural capital and impeding creativity and agency. This, in turn, can limit intangible community resources and threaten the collective capacity of the community (12, 14). While collective decision-making and leadership are important factors in community resilience, a sense of collective agency underpins any community initiative. Roberts and Townsend (12) argue such agency could have its origins in the development of cultural activity such as the influx into a community of cultural and artistic practitioners, which can have a spill-over effect fostering more diverse employment and economic outlets as well as bringing the community closer together. Steiner (14) reasons the initiation may also be provided from an external body, but that the community needs to be ready to undertake collective action. Linnell et al. (39) highlight community safety and security stem from a collective responsibility born by individuals within the community feeling they belong.

##### Collective leadership and engagement

Tudor et al. (37) elaborate on the role of creative skills in building community capacity through collective action. They outline the contribution of local groups of crafters in engaging and bringing together community members in the aftermath of the earthquakes in Christchurch, New Zealand, and in doing so provide an avenue for regaining control, improving economic and personal value, and developing connections with others and self (37). Congues (31) and Cinderby et al. (30) also explore the importance of skills as an avenue of engagement and in building collective capacity. Through skill-based activities the community can engage isolated community members, bridge gaps, and build and strengthen community cohesion. Programs that utilize tangible and intangible community resources to provide spaces, a sense of belonging and networks to run activities enable a more sustainable community that improves the collective leadership of the community, creates shared visions, and develops neighborhood assets for building resilience (30).

Likewise, de Schweinitz et al. (32) suggest programs that focus on the transfer of skills and knowledge and are activity-based provide opportunities for engagement and collective leadership. Action-based and outdoor activities provide key opportunities for at-risk groups to engage with others in the community. They also provide the opportunity for elders to mentor and exchange cross-generational dialogue with younger people, which provides support and purpose and builds collective capacity across generations within the community (32). Madsen et al. (33, 34) advocate leadership programs provide important skills that enable social connections and community cohesiveness, especially for isolated individuals or those who have not previously connected with the community. Madsen et al. (33) suggest collective leadership creates engagement through bridging social capital between members of the community, which is necessary for building resilience and human flourishing.

## Discussion

### Summary of Main Findings

Understanding the various types of “resilience” apparent in the literature, terms related to natural disasters were deliberately not included within the search strategy. Instead, the focus was on personal and community resilience more generally. Interestingly, more than 50 per-cent of the papers included in the review related to fast-moving (earthquakes and cyclones) or slow-moving (droughts and climate change) weather events. This may indicate a tendency to focus on resilience during adversity. In fact, funding for personal and community resilience is often easiest to obtain as part of disaster recovery programs. However, this focus can be somewhat misleading. Although adversity such as a natural disaster can offer opportunities for individuals and communities to adapt and transform, for the most part, resilience is generally developed in everyday circumstances. This holds for all members of a community, including older adults. While we will focus as much as possible on resilience relating to community-dwelling older adults, due to the paucity of research that targets this particular cohort, throughout this discussion we bring together the two strands of personal and community resilience outlined above and will draw on more general studies to explore: (1) actions that have demonstrated a strong association with growing resilience at both personal and community levels; (2) the circumstances in which transformation through adversity may occur; and (3) the caveat that local resilience may not be sufficient to change structural inequities but that certain actions can form the foundation for further advocacy and policy work. Overall, we will argue the re-framing of community-dwelling older adults as assets and sources of resilience within our communities. This does not take away from the need to meet specific needs for older adults during crises, but it does shift the narrative away from focusing on age-related deficits toward recognizing: (1) the extent to which older adults are already contributing to their own resilience as well as their communities' resilience; and (2) how these efforts could be enhanced through external facilitation.

#### Growing Resilience Through the Everyday

Keeping in mind the conceptual framework outlined in Figure 1, there are a number of personal and collective capacities and community resources that consistently appear in the literature as foundational to resilience. This review has identified self-esteem (21, 28) having a sense of meaning and purpose (21), having a sense of belonging (18, 19, 28) as well as developing well connected social networks and support (13, 18, 20, 22, 23, 25, 30, 33, 34). In addition, for certain groups such as First Nations Peoples, acknowledging the importance of community knowledge and cultural norms and practices is also important for resilience (29, 36). Community resources include facilities such as churches, videoconferencing facilities (such as those available in public libraries), Men's Sheds, and community meeting places (23, 28). Funding can also be considered as a community resource (14). Importantly, in order to truly appreciate the breadth of available resources, it is important to undertake a mapping of assets (39).

As such, interventions to increase resilience can be aimed across a number of levels toward increasing personal capacities, collective capacities, and/or community resources. While there has been considerable targeting of personal capacities within personal resilience research, particularly toward those who have psychological issues or disease, it does appear that interventions targeting general community resilience also spill over to increase personal resilience (9, 10). Those activities consistently cited as contributing to community resilience include:

- Volunteering (26);
- Collective action and participation (29–31);
- Collaborative decision making (36, 40);
- Community learning through collaborative action (33, 34).

Community-dwelling older adults play an important role in communities through their volunteering efforts. Seaman et al. (1) highlight volunteering's economic contribution to resilience, while Gibb (26) suggests volunteering helps older people to supplement gaps in aged support services. In many instances, volunteering provides older adults with additional social support and networks beyond their family, and an increased sense of belonging, meaning and purpose in life (22). Thus, increasing both personal and community resilience.

Activities that promote collective action and participation are important for increasing community resilience and act through a number of mechanisms. First, these activities necessitate mixing with other people, increasing social networks and support (1). Second, these activities help to build or consolidate community and cultural knowledge. Older adults are especially important resources of such knowledge in both Indigenous and non-Indigenous communities (1, 29, 36). Many of these activities require members to participate in collaborative decision making and the development of flat leadership governance structures which is important in developing group ownership and empowerment (1, 42). Collective activities also provide opportunities for collective learning as members negotiate everyday barriers and issues in undertaking their activities.

Whether these activities are related to crafting, making wooden tools, organizing indoor bowls, running adult education sessions or volunteering with a local charity, bringing community-dwelling older adults together on a regular basis to undertake everyday skill-based or social activities provides an opportunity to develop personal and community resilience that contributes to the level of general resilience apparent within the community. This is especially important in times of adversity. Thus, such activities are strongly reflected in the practice and policy recommendations we have put forward from this review (see Table 4). Participation, through volunteering, or in skills-based or social activities, that are accessible and affordable is fundamental to personal and community resilience in community-dwelling older adults.

**Table 4:** Practice, policy implications and areas for future research based on identified themes.

**Theme**	**Practice/Policy implications**	**Areas for future research**
Personal resilience	Encourage opportunities for community-dwelling older adults to volunteer	Establish the statistical relationship between volunteering, personal resilience and wellbeing Investigate factors that influence older adults volunteering
	Encourage opportunities for community-dwelling older adults to participate in community-based activities	Establish the personal benefits of participating in community-based activities Investigate factors that influence older adults participating in community-based activities
	Ensure community-dwelling older adults are aware of the opportunities available to them in the community	Investigate media avenues community-dwelling older adults use to find out about local community-based activities
Community resilience	Ensure community facilities are affordable to encourage community organizations and community-based activities for older adults	Undertake a cost-benefit analysis of community-based activities for older adults
	Should external facilitation be required to instigate community-based activity among older adults, ensure it is designed on an engagement-participation-empowerment basis to encourage ownership and collaborative leadership by community members	Investigate the role of external facilitation of community-based activity in rural communities and its short, medium and long-term impact on developing community resilience Explore the effectiveness of the engagement-participation-empowerment model in a variety of contexts
	Support and facilitate grassroots advocacy among community-dwelling older adults directed toward reducing inequities	Explore the effectiveness of grassroots advocacy in reducing structural inequities Investigate factors that influence older adult participation in grassroots advocacy
	Re-frame language and policies to reflect a strengths-based approach to resilience in community-dwelling older adults, recognizing the contribution older adults are making to the resilience of their communities	Evaluate the impact of re-framing language and policies related to community-dwelling adults to reflect a strengths-based approach to resilience within local government and non-government agencies

#### Reframing Adversity and Resilience

Adversity such as a natural disaster can provide an opportunity for growth by adapting or transforming the context in which the event took place (38). However, this is only likely to occur if there is already a high level of resilience within the community before the event (10, 25, 30, 43, 46) (Refer to the positive feedback loop in Figure 1). Fois and Forino (40) outline an example of transformation within an Italian village after a massive earthquake in 2009. Older adults worked with younger adults to develop an eco-village for temporary accommodation which led to the revitalization of other community facilities and assets. Yet, as Cinderby et al. (30), and Wilson (46) point out, adversity can also be the catalyst for a community ending up in a more stressed state which can lead to the withdrawal of services and depopulation. While the availability of economic and support services are important, it appears that the culture of the local community plays a significant role in how a community will respond to a crisis. Lyon and Parkins (41) compared two rural Canadian communities in the wake of mill closures and found that local culture was the key to how the community will transform. Seaman et al. (1) support this point. Here again, older adults can play an active role, particularly in First Nations Peoples communities where the elders are revered as keepers of cultural knowledge and ways (29, 36). This is where social narratives and the recalling of history, particularly in how the community has responded to difficulties in the past—stories that are held by older members of our communities—can contribute to community resilience (1). In effect, they can help reframe the disaster experience.

Counter-narratives to mainstream perceptions can also be useful in supporting resilience. Wood et al. (29) highlight the need for Aboriginal older women to counter the historical stories of deficit through stories of strength. Likewise, Seaman et al. (1) suggest older adults need to re-shape narratives of aging, acknowledging that they have often overcome many difficulties over their lifetime and have accumulated considerable personal resilience, as well as being able to contribute to community resilience. Attention should, therefore, be paid to the narratives surrounding older adults and their contributions to personal and community resilience, and consider these narratives in policy and research endeavors (see Table 4).

#### Caveat Regarding Local Resilience and Structural Inequalities

While the types of activities outlined above have been shown to increase resilience at a local level, it is important to emphasize this may not be sufficient to overcome structural inequities evident within the context. However, as argued by Ledwith (45), increased community activity that enhances participation and shared decision making can contribute by empowering local communities who may go on to advocate at broader political and social levels to eventually bring about structural change. Such action takes a long time but is at the heart of the settings approach within health promotion and community development (45, 47). Older adults can, and do, play important roles in such advocacy. Indeed, as our population ages and more “Baby Boomers” enter retirement, it is likely “gray advocacy” will become much more prevalent as a social movement.

Instigating the initial co-operative activity may occur organically through one or two people identifying an issue that is able to generate local support and eventually lead to broader action (45). Outside facilitation may also be necessary. For example, Steiner (14) outlines a project in Scotland whereby external facilitators worked with rural communities who were not taking advantage of funding to provide community facilities. Not all communities completed the projects, but this type of facilitation seemed to provide the impetus for some communities to start and then gather their own momentum, moving from a “stressed community” to a more vibrant one through a process of engagement, participation and empowerment (31).

## Limitations

There are a number of limitations associated with this rapid review. Due to time restraints, it was necessary to restrict the study parameters, and as such it is possible there are relevant studies that have not been included that are in languages other than English, published prior to 2013 or located in databases outside those searched. Furthermore, it was not always possible to identify the ages of participants in studies leading to the exclusion of potentially relevant studies. We also acknowledge the inherent limitations associated with the methodology, and that thematic analysis is useful for exploring the “what” and “how” however, does not provide insight into the strength of relationships.

As such, it is within the context of these limitations that we outline a number of potential practice and policy implications, and potential areas for future research (See Table 4). These primarily relate to: (1) encouraging community-dwelling adults to participate in community activities in order to collaborate with others; (2) promoting positive and healthy aging narratives by older adults, and about older adults; and (3) developing the evidence-base associated with such activities and resilience.

## Conclusions

This rapid review evaluated the current literature related to individual and community resilience in community-dwelling older adults to understand the status of resilience in this population, identify gaps, and make recommendations about effective interventions that promote improved individual and community level capacity. The review found personal resilience was based on positive reframing and agency, personal meaning and purpose, and acceptance and belonging. While at a community level, resilience was nourished by social empowerment, shared decision-making, agency, and collective leadership and engagement. The review highlighted the need to reframe how communities view older adults and shift the narrative away from focusing on age-related deficits toward acknowledging the economic and social contribution older adults make to the community through activities such as volunteering and the sharing of knowledge of history, culture, and skills. At a community level, activities that draw on these personal-level capacities to promote collective action and participation are important for increasing community resilience. The review also established that resilience is generally developed in everyday circumstances, therefore active involvement within communities needs to be encouraged within community-dwelling older adults. Developing active involvement will not only contribute to both personal and community level resilience, but will enable communities to prosper and flourish through adversity.

## Author Contributions

WM, MA, and MO designed the study, read and critiqued the articles, and wrote the paper. In addition, MO undertook the searches and MA undertook the thematic analysis.

### Conflict of Interest Statement

The authors declare that the research was conducted in the absence of any commercial or financial relationships that could be construed as a potential conflict of interest.
